# A Workflow for Building Computationally Rational Models of Human Behavior

**DOI:** 10.1007/s42113-024-00208-6

**Published:** 2024-08-15

**Authors:** Suyog Chandramouli, Danqing Shi, Aini Putkonen, Sebastiaan De Peuter, Shanshan Zhang, Jussi Jokinen, Andrew Howes, Antti Oulasvirta

**Affiliations:** 1 Aalto University, Espoo, Finland; 2 University of Helsinki, Helsinki, Finland; 3 University of Jyväskylä, Jyväskylä, Finland; 4 University of Exeter, Exeter, UK

**Keywords:** Computational rationality, Resource rationality, Modeling workflow, POMDPs

## Abstract

Computational rationality explains human behavior as arising due to the maximization of expected utility under the constraints imposed by the environment and limited cognitive resources. This simple assumption, when instantiated via partially observable Markov decision processes (POMDPs), gives rise to a powerful approach for modeling human adaptive behavior, within which a variety of internal models of cognition can be embedded. In particular, such an instantiation enables the use of methods from reinforcement learning (RL) to approximate the optimal policy solution to the sequential decision-making problems posed to the cognitive system in any given setting; this stands in contrast to requiring ad hoc hand-crafted rules for capturing adaptive behavior in more traditional cognitive architectures. However, despite their successes and promise for modeling human adaptive behavior across everyday tasks, computationally rational models that use RL are not easy to build. Being a hybrid of theoretical cognitive models and machine learning (ML) necessitates that model building take into account appropriate practices from both cognitive science and ML. The design of psychological assumptions and machine learning decisions concerning reward specification, policy optimization, parameter inference, and model selection are all tangled processes rife with pitfalls that can hinder the development of valid and effective models. Drawing from a decade of work on this approach, a workflow is outlined for tackling this challenge and is accompanied by a detailed discussion of the pros and cons at key decision points.

## Introduction

Computational models have played a central role in the field of cognitive science (McClelland, 2009; Shiffrin, 2010; Lake et al., 2017; Kriegeskorte & Douglas, 2018; Lieder & Griffiths, 2020). Models shed light on human behavior by precisely describing mechanisms that link hypothesized cognitive processes with environments and behaviors. Good models not only help develop theoretical understanding, but also produce useful predictions of future behavior, which can in turn be used in applied settings such as interactive AI. However, building such models, especially in scenarios that extend from controlled experiments in the laboratory to real-world behavior, has turned out to be challenging. One obstacle involves difficulties in capturing the extensive adaptivity that characterizes human behavior in the real world (Oulasvirta et al., 2022). To be applied practically, models need to account for how behavior changes as a function of beliefs, capabilities, goals, and the environment, which is, however, further complicated by the continual learning, adaptation, and exploration of individuals in their environment (Howes et al., 2023). Successfully tackling this challenge would advance efforts towards building computing systems that better understand people. For safer AI that collaborates better with people, we need models with control- and explanation-amenable causal mechanisms.

*Computational rationality* is a theory that has recently emerged as a candidate to address this challenge (Howes et al., 2009; Lewis et al., 2014; Gershman et al., 2015; Lieder & Griffiths, 2020; Oulasvirta et al., 2022; Howes et al., 2023).^1^ It draws together ideas from cognitive sciences and machine learning to predict adaptive behavior in artificial and biological agents. While the principle of rationality describes the ideal solution of a problem posed to a (unbounded) rational agent, computational rationality recognizes that biological agents also have computational limitations and provides a framework for describing agent behavior as a result of trying to make good decisions under computational constraints. Given our focus on human cognition in this article, the core assumption made by computational rationality is that our choices and behavior aim to maximize subjective expected utility; however, we are limited in that by bounds imposed not only by the “external environment,” but also an “internal environment” comprising of our cognition and our bodies. The external environment here refers to elements such as our physical context, the task being carried out, or the devices we interact with that shape our possible behaviors, while the internal environment includes latent psychological constructs such as perception, attention, and memory that may have capacity limits. Observed behavior is then considered an emergent consequence of rational adaptation to the totality of these constraints.

While there are several ways of instantiating computational rationality (Lewis et al., 2014; Lieder & Griffiths, 2020; Icard, 2023), our article is concerned with a framework that uses partially observable Markov decision processes (POMDPs; Sondik, 1971) to capture computational rationality as arising due to the partial observability of the external environment and internal environments. POMDP-based models are theoretically sound and flexibly allow for the incorporation of a variety of models within them, including those based on deep learning, which can be useful in capturing the complexities of real-world environments and behavior across a wide range of contexts. Methods from reinforcement learning and deep reinforcement learning can be then used for solving the POMDPs (i.e., finding approximate policies that predict observed behavior). As these models are related to practices in both, cognitive science and machine learning, we will be using terminology from both disciplines in the article. We introduce and clarify key terms when they come up to aid in comprehension.

**Fig. 1:**
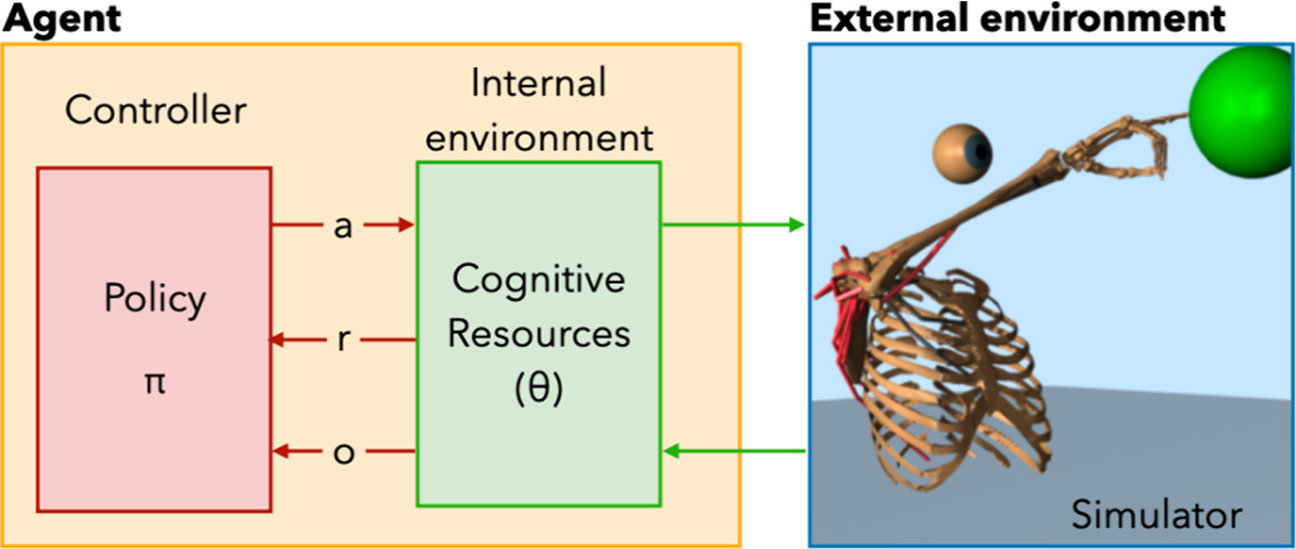
Computational rationality assumes that people adapt their behavior to maximize expected utility under cognitive bounds. To build a computational model that instantiates this theory with reinforcement learning, a policy ( $\pi$) needs to specify a decision problem where a policy ($\pi$) controls which action (*a*) to take in light of observations (*o*) to maximize rewards (*r*). Whereas standard reinforcement learning-based models applied in ML are situated directly in the external environment, computationally rational models of human behavior are only “yoked to it” via their internal environment, that is, cognition. Modeling the decision-making problem, including the internal environment, as well as its parametric variability across individuals ($\theta$), is a challenge in cognitive science

The computationally rational models that we build with reinforcement learning have some basis in traditional *cognitive architecture* approaches (Laird et al., 1987; Anderson et al., 1997; Bekolay et al., 2014), but vary greatly in terms of how they model adaptive behavior. Cognitive architectures assume that everyday tasks recruit multiple cognitive capacities that need to be carefully “architected” into a model (Card et el. 1983) and consequently model internal capacities as resource-limited modules; the architectures include a “processor” that directs the flow of information between modules based on a “cognitive program” or a set of “production rules.” Although cognitive architectures have made significant progress with respect to understanding cognition, they have sometimes been brittle as predictive models because their “programming” was often hand-coding them via “sophisticated guessing” (Shiffrin, 2010). Every time a different situation or individual needs to be studied, the vast rule base needs to be manually updated, which can be time-consuming and contrary to its vision of producing generally applicable models of behavior. Computationally rational models, on the other hand, capture the essence of cognitive architectures within the “internal environment” of a reinforcement learning agent (Lewis et al., 2014). Adaptive behavior then emerges as a consequence of bounded rational policy optimization. Boundedly optimal behavior can be derived for different situations based only on policy optimization. When building a computationally rational model, one specifies the environment and the agent’s goals and characteristics; the actual behavior in a given situation, however, emerges via a *policy* ($\pi$) optimized to these bounds. In practice, this policy is approximated by machine learning methods, particularly from reinforcement learning. Computationally rational models are also generative in the sense that they create stepwise simulations of an agent’s behavior in a given setting, rather than merely predicting summary statistics of empirical data. This is made possible due to parameter inference that it allows at both the individual and the group level, depending on the context of application. During the last decade, the theory has been successfully applied to data from increasingly realistic everyday tasks, including typing (Jokinen et al., 2021a), driving (Jokinen et al., 2021b), visual search (Radulescu et al., 2022; Jokinen et al., 2020; Todi et al., 2019), multitasking (Gebhardt et al., 2021), menu interaction (Chen et al., 2015), and affordances (Liao et al., 2022), among others including (Belousov et al., 2016; Chen et al., 2021). We note that computational rationality models built with reinforcement learning are distinct from reinforcement learning-based models of human learning such as Pavlovian learning (Rescorla, 1972; Zhang et al., 2020) and error-driven learning (Seymour et al., 2004; Sutton, 1988).

Computationally rational models are based on a specification of a decision-making problem that an agent faces. At the heart of this formulation are three elements, outlined in Fig. 1: (i) the internal environment of the agent (that is, the cognitive resources and processes involved), (ii) a reward signal representing what is important to the agent, and (iii) the external environment in which the agent operates. Moreover, the modeler needs to decide the internal representations through which the agent is observing the world. This dictates how external stimuli are transformed into an internally represented state abstraction or a belief that informs the agent’s choice of how to act. The internal environment can be represented in a variety of forms ranging from simple mathematical models to end-to-end machine-learned modules, and it can also take various architectural forms, such as hierarchical (e.g., Gebhardt et al., 2021) and modular architectures (e.g., Jokinen et al., 2020). The place where the approach breaks decisively from cognitive architectures is in its formulation of a sequential decision-making problem. When the specification of the agent adheres to the assumptions of a Markov decision process (MDP), machine learning methods can be used to estimate an optimal policy. Reinforcement learning (RL), in particular, has been one of the most widely used frameworks used to this end (Sutton & Barto, 2018). It learns an optimal bounded policy through trial and error.

Despite the success of computational rationality models framed as POMDPs, producing such models in a valid and practically applicable manner is not straightforward. The usefulness of the eventual model critically depends on several modeling decisions that are made while building them from (i) the initial steps of specifying the rewards and cognitive resources to implement in the model, to (ii) formulating the task as a sequential decision-making problem, and (iii) using RL methods to approximate the optimal policy. The impetus for this paper comes from numerous struggles and dead ends encountered in grappling with computationally rational models of everyday behavior over a decade of research. It is a considerable hurdle to produce such models; the validity and applicability of these models also critically depend on choices made on the way. While there are excellent guidelines for developing other types of computational models of behavior, such as Bayesian statistical models (Gelman et al., 2020), decision-making models (Wilson & Collins, 2019), and reinforcement learning (Zhang et al., 2020; Patterson et al., 2023), computational rationality comes bundled with many unique challenges and considerations. These questions are among its complicating factors: How to choose what to handle via RL and what learning should occur with a cognitive resource? Which measures of model quality are meaningful? How does one specify the reward functions? How does one perform model checking when the models use interpretable theory-based components as well as tractable data-driven components? How much value do metrics for model parsimony offer for handling model selection in such scenarios?

**Fig. 2:**
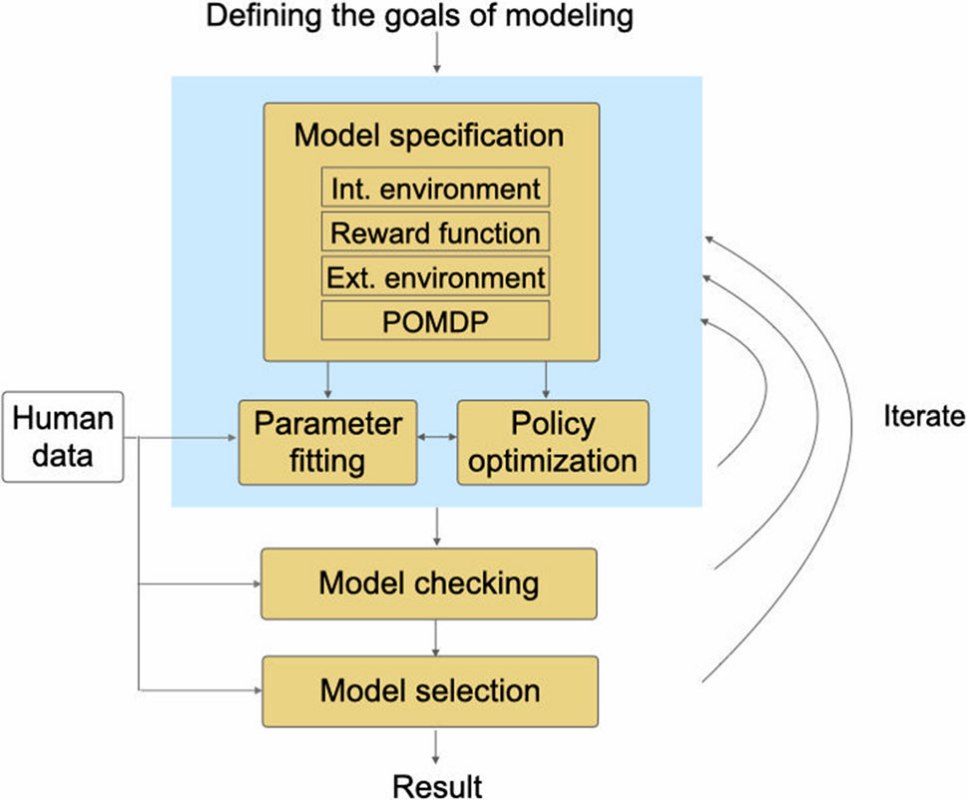
A summary of the model building steps considered in the workflow for developing computationally rational cognitive models with POMDPs. The model building workflow is tangled and can proceed in several different directions along the arrows shown, and it is iterative with a series of model revisions based on model checks. Models often start with simple specifications and they are gradually improved in quality and complexity until they capture the key elements of empirical human data and satisfy the modeling goals

This paper fills this gap by laying out a workflow framework that is able to guide modelers interested in computational rationality (diagrammed in Fig. 2). A key aspect of the proposed workflow is its iterative stepwise structure. This incorporates steps that are vital for cognitive modeling (model specification) but also elements necessitated by ML structures (policy optimization and parameter tuning), alongside general stages in computational modeling (checking, comparison, and selection of models). Since machine learning approaches are used in this form of cognitive modeling, our article will use relevant terminology from these disciplines.

Workflows are beneficial in and of themselves and complement the progress achieved through the development of new tools and software in the field. For instance, powerful software libraries in statistics and machine learning have made it easier to develop complex models of human behavior. However, it has become equally possible to build models without putting much thought into them (Hullman et al., 2022; Kapoor et al., 2023) or to use methods such as deep learning when there is a paucity of training data, which is the scenario for almost all cognitive science experiments and user modeling approaches. Such practices often result in models that are poorly specified—that is, untrustworthy models that make patently inaccurate assumptions about the world, and are neither useful for insight and understanding nor for prediction and generalization. Misspecified models may fit observed data well but are fragile and break down under even the slightest variation in the environment. However, even though there are overheads to following a model building workflow, the many implicit sanity checks, diagnostics, and tests can increase the quality, usefulness, and robustness of models. Models developed through a structured workflow tend to be more carefully thought out, tested, and reported upon. In addition, anchoring modeling choices to a workflow is also one way to be transparent about the modeling process. Hence, we conclude that principled workflows rank among the keys to better cognitive science, conferring benefits for validity, reproducibility, and accumulating valuable scientific results. However, we must stress that workflows are better treated as guides rather than rigid prescriptions, to leave flexibility for situation-specific modeling contexts and goals.

A considerable portion of the workflow is related to specifying the agent’s internal environment. Introducing such causal underpinnings is especially beneficial in cases where explanation, control, and prediction are crucial. However, this simultaneously renders computationally rational models vulnerable to misspecification: the assumptions and processes articulated by the model might yield poor approximations of reality or turn out to be simply wrong. This is why our workflow’s iteration incorporates such steps as model checking, which studies have linked to reduced misspecification and greater robustness across multiple model classes and modeling domains (Box, 1980; Wilson & Collins, 2019; Gelman et al., 2020). Taking into account these considerations, we pursue the following objectives in designing the workflow:

1. Pre-specifying objectives: Articulating a domain of behavior in advance (“This is what we aim to model”) and specifying both the key phenomena in the data and metrics for success is essential for tracking the progress of the modeling effort. Although all modeling is explorative and subject to change accordingly, working with clear goals from the outset may safeguard against unhealthy practices such as declaring modeling goals opportunistically after the fact and without validating the model for them appropriately.
2. Separation of policy learning and parameter fitting: Some free parameters of a computationally rational model describe the policy, while others characterize the individual. Though the two often get optimized jointly, they are conceptually distinct.
3. Model checking and validation. These practices increase the quality of models by detecting overfitting and ill-defined structures and parameters.
4. Scaling up iteratively: Getting the model right the first time is nearly impossible, in that success rides not only on theory-based insight but, regrettably, also on luck (e.g., the policy must converge and depend on random-number seeds). Therefore, we recommend deliberately planning for iterative model building that starts with a minimal goal set and expands in each iteration towards the full scope.

Our presentation begins with a review of prior attention to workflows for this class of modeling. Against that backdrop, we then explicate the proposed workflow, with a focus on each step’s major decisions. The discussion pinpoints key considerations and highlights central choices specific to computational rationality. Because of the emerging nature of this area, many of the discussions we offer are meant to expose their respective pros and cons choices rather than prescribing an approach.

To anchor our discussion in a practical case, we present a running example from recent modeling: the gray box at the end of each section addresses an attempt to extend state-based models of human typing behavior (Jokinen et al., 2021a) on touchscreens to a pixel-based agent (Shi et al., 2024). Typing exemplifies the challenge well since it involves adapting eye–hand coordination in accordance with external bounds (from design, intelligent text entry, etc.) and internal ones (e.g., working memory capacity and noise levels). The agent, limited by partial observability (foveated vision), must decide where to direct attention at any given time: the keyboard, the text display, the backspace key, or some other element. To be useful, a model of typing behavior should be able to predict the effects of changing conditions counterfactually (Oulasvirta & Hornbæk, 2022). For instance, how will the user’s typing strategy and performance change if an intelligent text entry system enters the loop, when the user cares less about errors, or if there is a switch in keyboard layout? Moreover, to capture individuals’ differences, the typing model needs to be tunable for individual-level data; this implies that we should be able to invert them when given humans’ data.

## Background: Workflows for Rationality-Based Models of Human Behavior

In the context of modeling, a workflow is a systematic approach for building valid, reliable, and useful models through a sequence of iterative model building steps. A workflow captures both formal knowledge and tacit knowledge into clear and actionable guidelines. These guidelines may draw from numerous types of knowledge: theoretically proven solutions, best practice identified in the literature, an experienced colleague’s wisdom, awareness-raising reflection, etc. A workflow provides a systematic framework for navigating the garden of forking paths of modeling choices to produce high-quality models.

The need for workflow-based model development is accentuated in cases that combine the two cultures of modeling (Breiman, 2001)—where our model simultaneously combines generative processes informed by psychology theory with those learned from data using machine learning—computationally rationality models that use reinforcement learning are an example of such a hybrid approach.

Workflows for computational models often distill them into four main steps: (i) model specification, (ii) model fitting, (iii) model checking, and (iv) model selection. Each step comprises sub-steps, diagnostic checks, and best practices for model building. In this article, we do not deal with data collection as a part of the modeling workflow, even though data are important for modeling. We assume here that data has already been collected in a valid and rigorous manner and focus on the iterative model building workflow given such data.

While the general objective behind modeling workflows (e.g., see Schad et al., 2021; Zhang et al., 2020; Wilson & Collins, 2019; Gelman et al., 2020; Schad et al., 2022; Patterson et al., 2023; Grinsztajn et al., 2021) is to enhance the quality of scientific research, their specific steps and emphases differ, reflecting the distinctive goals and methods of the respective domains. This article specifically targets the development of computationally rational models instantiated using reinforcement learning.

This paper extends workflows for rationality-based models of human behavior to the case of computational rationality. In rational analysis (Anderson, 1991; Chater & Oaksford, 2000), the idea is that cognition adapts to requirements of the environment. Accordingly, the modeling centers on developing agents that produce optimal behavior for a given task and the statistical structure of its environment. Lieder and Griffiths (2020) recently sketched out a high-level workflow for resource-rational (computationally rational) analysis (see also Howes et al., 2009), splitting it into five steps:

1. Start with a functional description of an aspect of cognition
2. Articulate the algorithms for cognition, with cost and utility values
3. Pick the algorithm that optimally trades off cost and utility
4. Compare with empirical data
5. Iterate until satisfied with the result

While this outline provides a good sense of the general flow, the emphasis is on understanding a particular aspect of cognition, and situating these perspectives in Marr’s levels of analysis framework (Marr & Poggio, 1976), rather than modeling behavior in complex, everyday contexts. Consequently, it overlooks the detailed steps necessary for practical modeling, especially in defining decision-making problem and estimating the (boundedly) optimal policy of the agent (Russell & Subramanian, 1994). There is a gap in integrating these cognitive and rationality-focused aspects of computationally rational models with the general computational modeling building frameworks in cognitive science, machine learning, and statistics. Our article bridges this gap by presenting a comprehensive workflow for developing computational rationality models using reinforcement learning. This workflow is informed by our experience developing such models and integrated with established modeling approaches; it aims to avoid common pitfalls and improve the validity and effectiveness of our resultant model.

No known workflow met all of our needs when we began work on the computationally rational typing model. Such development requires understanding of rational analysis, deep reinforcement learning, and computational statistics.

## Defining the Goals of Modeling

Before starting to build a model, it can be very useful to explicate the goals of modeling and plans for achieving them. Preregistration of modeling goals and procedures have been promoted for their possible reproducibility benefits (Lee et al., 2019; Hofman et al., 2023; Kapoor et al., 2024), but we consider it useful for guiding the modeling workflow and decisions made along the way, for model evaluation against our articulated objectives, for transparency, and improved communication behind the motivation of choices made. The general aspects of the model worth articulating ahead of model building include the following:

1. Purpose: Why is the model being created?
2. Scope: What theories, behavior, and data are covered, and to what extent? Which human behaviors do we want to reproduce, under what types of theoretical assumptions, and in line with which data?
3. Baselines: What models can address the relevant goals and scope?
4. Metrics: Which metrics are best suited to evaluating the model’s quality?

### Clarifying Purposes

Developing a model should start by laying out the general goal for the model: what is its purpose, and why does it need to be created? For example, the goals for modeling human behavior may be theoretical—to understand and explain general mechanisms underlying human cognition across varied contexts, and advance scientific knowledge. Or the goals may be practical, where they are relevant for a particular application setting, where aspects such as predictive performance and computational efficiency may override explanatory considerations. Each modeling endeavor may have different objectives and purposes, and it helps to articulate them before building a model. Articulating goals upfront can serve as sanity checks to keep in mind as we iteratively develop a model and make decisions throughout the model building process.

### Setting the Scope

After explicitly stating the overarching purpose, we need to define the model’s intended scope. There are three aspects to this: (i) theory scope, (ii) behavior scope, and (iii) data scope.

The first involves specifying the psychological assumptions in terms of modeling decisions. For example, if we are interested in modeling visual search of natural scenes, we might decide to include assumptions about foveated vision and how the ability to detect visual features drops off in peripheral vision (Kieras & Hornof, 2014). Setting the theory scope involves picking out the relevant aspects of cognition that will form the foundation of the model: we might choose to focus on those related to attention, memory, perception, or goals and motivation.

Secondly, we must specify the behavior scope. Involving the empirical phenomena and environments that the model should cover, the behavior scope delineates the conditions in which the model is assumed to operate and how it does so. Often, when we model data collected in an experimental study or a set of studies, the behavior scope can be dictated by our theory scope, experiment settings, conditions, and behaviors—the types of stimuli presented, the objectives given to the participants, the nature of responses elicited from them, etc. The practical application context can be another determinant of the behavior scope. For instance, we may have in mind a model of typing that makes predictions for novel keyboard layouts. In that case, the behavior scope should cover such additional keyboards.

Finally, any data and data patterns (i.e., behavioral patterns) that we want the model to account for fall under data scope. Examining and understanding the data is important for gaining insights into the data patterns we would like the eventual model to predict and explain. Exploratory data analysis (EDA) refers to such a process of examining the data and its variables by various means such as visualizations and descriptive statistics (Wilson & Collins, 2019; Gelman et al., 2020). The objective here is to concretize the modeling goals by grounding them within the data at hand. EDA can also act as a check on the quality of the dataset and allow us to examine if there are any outliers or critical errors during data collection which reduces the usefulness of the data. Also, one has to assess the quality of the dataset, pinning down factors such as noise and whether there are outliers or missing data. Even more critical is the need to visualize the patterns identified as belonging to the data scope. Tools such as scatterplots, violin plots, histograms, and animations can serve this purpose. These equip us with a comparator that enables better gauging the model’s behavior at the model checking stage.

### Defining Baselines

Choosing meaningful baseline models is important for demonstrating the value and novelty of our proposed model in a valid manner, within the context of existing approaches. Hence, once interesting data patterns have been identified and prioritized, it helps to consider existing models from the literature that are relevant to the task and patterns we consider important. Predictive performance is a key indicator of a model’s effectiveness, and it is hence a common practice to choose current state-of-the-art approaches in the field as a baseline against which to compare our eventual computationally rational model. In some cases, theoretically interesting alternatives may serve as more natural baselines as they allow us to contrast the implications of different assumptions on model behavior. In addition to testing the effectiveness of our eventual model, these baselines can also inspire ideas about what to incorporate in our model and serve as a basis on which our model can be iteratively built. The initial goal may be to perform at least as well as the baselines, with the ultimate aim being to surpass their performance. If the baselines we select are available in code, we recommend trying them out against the visualizations produced in the steps above.

### Establishing Model Performance Metrics and Diagnostics

Model performance metrics are quantitative measures that can be used to assess how well a model performs against its objectives. For example, common metrics to measure predictive performance against observed data can be metrics such as accuracy, precision, and root mean squared error. In addition to the broad prediction metrics, we may also be interested in how well predictions capture qualitative data patterns that we had declared as important to capture (e.g., in visual search, a pattern of search times being longer when the target and distractors are of the same color). We recommend mapping out key metrics that align with the modeling objectives to ensure that the workflow remains focused on the modeling goals and to measure progress on these objectives.

Diagnostics are measures that can identify problems that arise during modeling and often provide insight into the inner workings of the model. For example, feature importance (Casalicchio et al., 2019), which indicates the features most influential for a model, can be diagnostic to determine if any neural network components used in the model are emphasizing sensible features or not; sensitivity analysis, which evaluates the model’s performance under different settings, can provide insights into the model’s robustness and sensitivity to different assumptions. It is recommended to think of these main metrics and diagnostics up front to be used throughout the various stages of modeling. We offer several examples in the course of the discussion below, but in the ideal case, one should select metrics that reflect the scope well and pick diagnostics are maximally informative with regard to how well the modeling is progressing.

### Planning the Project

The complexity of models in this domain makes it nearly impossible to create a valid model that fulfills all modeling goals in one attempt. An iterative modeling workflow that starts with simpler accounts for observed data has several benefits. Simpler models have fewer components and allow us to better diagnose and debug modelling issues as they come up. An iterative workflow also makes it easier for modelers to gradually improve their understanding of observed behavior and model behavior. Such an iterative workflow structure can be strategically planned, and we turn to the notion of a research ladder, describing a few milestones for the modeling process in progressively higher fidelity. Because policy optimization is often a time-consuming step, it also makes sense to work with simpler versions of the problem at first and ensure we are on the right track before increasing complexity. Our planning typically defines ladders with 3–4 steps, each for matching (i) a certain human phenomenon at (ii) a certain level of fidelity (e.g., trends only or absolute numbers). On some occasions, one may choose the alternative strategy of starting from a higher rung of the ladder from a known and well-understood model, and then stepping down by simplifying its assumptions until we come to a better understanding of how the original model needs to be changed; thereafter, one can scale up the ladder again until the changes yield satisfactory predictive performance and satisfies our modeling goals.

The primary goal set for the touchscreen-typing model was to gain insight into the behavior of human typists and subsequently use the model to evaluate keyboard designs. Accordingly, we specified that the model should coordinate finger and gaze movements for typing phrases on touchscreens in a human-like manner. We wanted to improve accessibility by simulating users who vary in capabilities and predicting design changes’ impact on their performance. We set the scope for the typing model by way of three considerations.

Firstly, the model should have the capacity to replicate main tendencies in an empirical dataset of typing (Jiang et al., 2020). Its policy should closely align with human data and replicate key metrics and phenomena documented in the paper. Secondly, typing patterns vary considerably between individuals, with some displaying faster typing, some being slower, some making more errors, and some proofreading more. It is vital to model these individual differences, especially for applications that support special user groups. We aimed to replicate the distribution of behavior that reflects a wide range of user populations, as illustrated in the figure below. Finally, we sought a model that performs well not only for the specific keyboard it was trained on but also with previously unseen keyboard layouts. It had to function well across a wide range of keyboard designs, layouts, and intelligent features. 
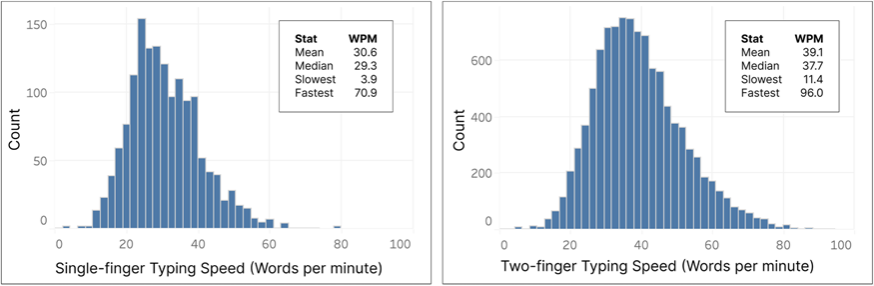


To ascertain how well the model matches real-world eye and finger movements in terms of typing speed, error correction, and proofreading, we chose six representative metrics from work by Wobbrock (2007); Arif and Stuerzlinger (2009), and Feit et al. (2016): words per minute (WPM), inter-key interval (IKI), amount of backspacing, error rate, number of gaze shifts, and gaze-on-keyboard time ratio. We selected the latest state-based RL model (Jokinen et al., 2021a) as our baseline.

To scale up our modeling efforts, we developed a research ladder with a simple finger-movement pattern as its lowest rung. More complex eye–hand coordination patterns were higher up the ladder. After attending to finger behavior, specifically typing speed (represented by WPM and IKI figures), we added the layer of difficulty from error-correction strategy, by incorporating error rate and Backspace presses. Finally, we modeled gaze behavior, which involves more complicated mechanisms connected with proofreading strategy (gaze-shifting, etc.).

## Model Specification

In the implementations of computational rationality via POMDPs, we model humans as agents interacting with their external environment through the lens of their own cognitive limitations, such as bounded rationality, limited attention, and imperfect memory; models are conceptually partitioned into an internal and an external environment, as Fig. 1 illustrates. The **external environment** represents the task environment encountered by the agent. For example, a model addressing a car-driver’s external environment might contain simulations of the steering wheel, the pedals, and the vehicle as a dynamic object in traffic. The **internal environment**, in contrast, encompasses the cognitive processes internal to the agent, including the generation of observations and reward signals. This internal/external division is critical. The agent does not interact with the external environment directly; instead, engagement is with an internal representation of the world, shaped by the agent’s cognitive processes. The internal representation allows the agent to interact with its environment in a way that is computationally feasible for everyday tasks and situations (Lieder & Griffiths, 2020). A partially observable Markov decision process, or POMDP (discussed below), formalizes how these environments are available to the agent. It translates the underlying state of the external world, which is only partially observable, into a form that the agent can perceive and act upon.

### Specifying the Internal Environment

While optimal adaptation exclusively to the design of the external environment would be an outcome of (unbounded) rational behavior, optimal adaptation to the internal environment results in a computationally rational behavior that adapts not only to the external environment but also to the agent’s computational bounds imposed by its cognitive limitations. The agent’s design typically involves cognitive processes such as memory, perception, and other faculties through which it perceives and acts in its world (the external environment). The representations produced cover a spectrum from simple noisy perception to complex representations of the agent’s goals, beliefs about the external environment, tool systems usable in the external environment, and other constructs. Other factors captured within the internal environment might consider fatigue, stress, and similar physiological phenomena.

For a computationally rational model, hypotheses about cognition are encoded in the internal environment. There are various ways to implement the assumptions for the internal environment: via symbolic approaches, Bayesian inference, rules, or neural networks, among other mechanisms. Modelers express the bounds of the internal environment through the cognitive model’s parameters. This step goes by the term “parameter specification” and involves “fixing” relevant parameters to a particular value so as to codify an assumption we are making about the agent or environment. For example, we may want to fix or specify the parameters of not only a known external environment, but also the psychological assumptions about the agent such as a particular working memory capacity that is generating observed behavior. Parameters are typically fixed or specified based on prior knowledge, known empirical findings, or theoretical considerations. With Bayesian methods, it is possible to specify degrees of beliefs in parameters’ values by constructing a prior probability distribution (Mikkola et al., 2023) and update these prior beliefs into a posterior probability distribution given data. Bayesian approaches to computationally rational POMDP models are currently uncommon, but quite possible—for example, Kangasrääsiö et al. (2017) used a Gaussian prior for plausible fixation durations in their menu search model; Shi et al. (2024), placed a uniform prior distribution over a range of plausible parameter values, and explained in the typing example. Also, see Aushev et al. (2023). However, Bayesian approaches are not yet common in the field, due to the computing costs when using a distribution over parameter values. After appropriately specifying any parameter’s value or prior, it is also important to identify which parameters vary at different levels: individual-level parameters that vary between individuals, group-level parameters that vary between groups, and population-level parameters that remain constant across the entire population. While we specify theoretical assumptions by parameter specification or the specification of their priors, an agent may also be designed to perceive some of those variables (e.g., its stress level) and even manipulate them (choosing what to store in working memory, etc.) as it interacts with its environments.

### Designing the Reward Function

The second step in model building involves specifying these internal reward signals. This reward can be defined based on one of two perspectives: (i) the reward signals are considered to be generated by the external environment, and these rewards are a proxy for internal rewards, or (ii) it can be defined internally based on representations in the internal environment. The former approach is the more straightforward—from this point of view, rewards indicate the goodness of any given action as defined by the task, and the agent aims to maximize the long-term rewards even if it is done in a computationally rational manner taking into account constraints posed by both environments.

From a psychological perspective, the rewards are always internal. External rewards can sometimes be a proxy for internal rewards; but, when this assumption fails, we need to explicitly specify a psychological mechanism for how humans generate internal rewards (Lee et al., 2023). Human policies are not completely determined by the task environment—we use curiosity and intrinsic motivation to explore the world even when it lacks explicit rewards.

When reward functions are specified as a feature of the internal environment, we need to investigate the learned policies to understand the behaviors that these subjective rewards produce. That is, the agent has goals that it is externally expected to achieve (e.g., pressing a particular key), however, the reward function may, in fact lead to other types of behavior (e.g., pressing no keys if mistakes are heavily penalized). The way to verify this is via running simulations of the model and examining whether the reward function is leading the agent towards the desired objectives.This type of verification differs from model checking in the sense that we are mainly ensuring that the agent can technically achieve the objectives it has been specified.

When a reward function is not specified accurately, we may observe a phenomenon termed “reward hacking” (Clark & Amodei, 2016), where the agent discovers unforeseen shortcuts to achieve high rewards without actually learning the desired behavior. As the agent and environment increase in complexity, it becomes harder to design reward functions that do not reward hack. Recent research provides subworkflows to prevent this phenomenon, for example, by detecting anomalous or aberrant policies (Pan et al., 2022) or by iteratively shaping reward functions (Gajcin et al., 2023).

### Specifying the External Environment

The third step is to specify the external environment. It can represent a task environment, for example, a setting where a user is supposed to type, or it can be an open world like Minecraft. Practically, however, projects often start with a gridworld or similar “toy environments”, which may help focus on the bare essential aspects of the problem at hand. There is a wide variety of software available for creating and working with external environments ranging from physics simulators (e.g., MuJoCo) to agent simulators (e.g., Sumo and TFAgents), as well as software emulators (e.g., AndroidEnv), among others. What matters is that the software that is used is (i) fast enough to permit training policies and (ii) allows interaction: the internal environment must be able to provide data to the perception and enact the agent’s actions.

### Defining the Agent’s Decision-making Problem

The elements defined in the previous sections, together, specify a POMDP: a sequential decision-making problem under partial observability. The POMDP is defined by the tuple$(\mathcal {S}, \mathcal {A}, \mathcal {O}, T, O, R, \gamma )$ where $\mathcal {S}$ is the state space, $\mathcal {A}$ is the action space, $\mathcal {O}$ is the observation space, $T: \mathcal {S} \times \mathcal {A} \times \mathcal {S} \rightarrow [0, 1]$ is the state transition probability function, $O: \mathcal {S} \times \mathcal {A} \times \mathcal {O} \rightarrow [0, 1]$ is the observation probability function, $R: \mathcal {S} \times \mathcal {A} \rightarrow \mathbb {R}$ is the reward function and $\gamma \in [0, 1]$ is the discount factor. Given an action $a \in \mathcal {A}$ and current state $s \in \mathcal {S}$, the agent transitions to a new state $s' \in \mathcal {S}$ with probability $T(s, a, s')$, receives a reward *R*(*s*, *a*), and observes $o \in \mathcal {O}$ with probability *O*(*s*, *a*, *o*).

The POMDP formalism is designed to align with the principles of computational rationality. It recognizes that an agent’s knowledge of the world is often incomplete and models this uncertainty through partial observability. The state transition and observation probabilities, along with the action space, encapsulate the concept of a dynamic and probabilistic world, which an agent needs to navigate with its limited cognitive resources. Cognitive resources can be flexibly represented within this framework as part of the transition function, observatin, and/or actions. Finally, agents act to maximize the sum of discounted future rewards, reflecting the process of utility optimization.

### A Note on State Abstractions

A critical design choice concerns the observation function. Because the full state of cognition (the internal state) is complex, a computationally rational agent typically only observes part of it. While partial observations hold clear value—they make exploration and policy optimization significantly easier—this simplification loses information, some of which might be relevant for the task. The choices made in defining the observation function carry a risk of errors compounding over a long planning horizon (Talvitie, 2014; Ye et al., 2021; Starre et al., 2022). Prior work offers some guidance for creating effective observations. Theoretical research has established that observations can safely aggregate across states that are identical in their reward and transition probabilities; likewise, states with identical *Q*-values under the optimal policy can be aggregated (Li et al., 2006). The literature also deals with stronger aggregations, which combine more states together. However, policy optimization such as Q-learning on these aggregated state spaces is not always guaranteed to produce a policy that is optimal (under the original state space) (Li et al., 2006). Approximate versions of the aggregation criteria are also available to aggregate all states with reward values and transition probabilities that are not identical but within $\varepsilon$ of each other. Though approximate aggregations introduce some error, the resulting value loss is polynomially bounded for specific aggregation strategies (Abel et al., 2016).

The model we built is a computationally rational typist (CRTypist). We designed its internal environment to specify several crucial bounds of human vision, motor control, and working memory. That environment functioned as a bridge between the agent and the touchscreen. The **Vision** module processes information as pixels from a small focal area and blurred information from peripheral vision. **Finger** simulates pointing to a specific pixel on the screen, which may entail position errors stemming from rapid movement or lack of visual guidance. Finally, **Working Memory** stores information from the vision and finger movements, assessing the typed text while accounting for uncertainty due to time decay. All components of the internal environment feature separate parameters representing their capabilities. The agent’s controller does not interact with the touchscreen directly. It interacts with the internal environment by setting goals for the eye and finger movements. Pursuing these goals, the Finger and Vision modules point to a specific position on the touchscreen. 
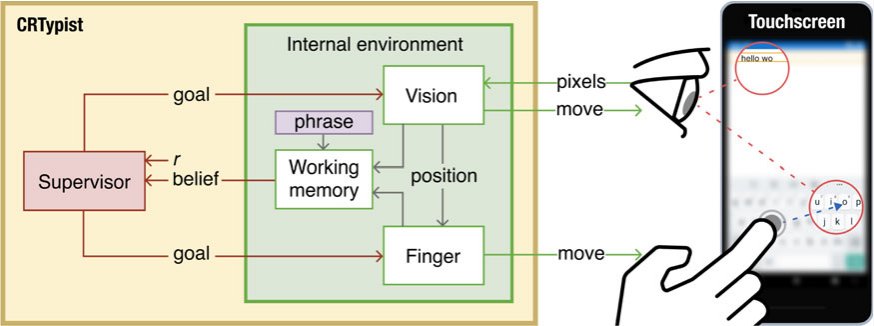


We design the reward function by considering the speed–accuracy tradeoff. In essence, the reward received is a compromise between how accurately and how quickly one can type. The reward is given at the end of each episode, upon pressing the Enter key. The mobile touchscreen served as the external environment. We captured pixel-representation images from a software emulator to obtain the visual information from the keyboard and text display both.

Within the framework outlined above, we formulated the POMDP thus:

- Within the state space, $\mathcal {S}$, a state $s_t$ consists of the pixel representation of the touchscreen display at timestep *t*, including both the keyboard and the text area.
- In the observation space, donated as $\mathcal {O}$, an observation ($o_t$) consists of the beliefs from the working memory (which stores information gathered from foveal and peripheral vision).
- An action *a* in the internal environment sets target goals for both vision and finger—within the action space, $\mathcal {A}$. It leads to the behavior the typist can execute on touchscreens—gaze movements, tapping with a finger, etc.
- In the context of typing, the reward function $\mathcal {R}$ is expressed with a speed–accuracy tradeoff in the cognitive model of the mind: the goal is to type correct target phrases as quickly as possible.

## Policy Optimization

As with any other class of computational models, the performance and predictions of computationally rational models are influenced by how the models are configured. We can divide the model-configuration variables into two types: hyperparameters and model parameters. Hyperparameters are variables that are external to the model and whose values are not learned from the data but are specified by the modeler to determine how the model learns. Model parameters, on the other hand, help to form the internal structure of the computationally rational model, and are inferred or estimated based on the data to which the models are fitted. Appropriately specifying model parameters and hyperparameters is essential for obtaining valid inferences and predictions from the model.

Model parameters can be further divided into three categories: (i) the parameters of the internal environment that have a clear interpretation and a theoretical grounding, (ii) the parameters that represent the optimal policy of the POMDP, and (iii) parameters of the external environment, such as those representing task distributions.

The parameters of the internal environment are psychologically meaningful parameters that may either be specified to reflect modeling assumptions (see previous section), or they can be estimated from observed data through parameter fitting (see next section).

On the other hand, the policy parameters parameterize a neural network that represents the policy and, therefore, are difficult to interpret. Given some initial values for the POMDP, a policy repeatedly directs the internal environment and is given a reward. Over many episodes, the policy is trained to maximize the reward received until a given stop condition is met, which is indicative of satisfactory level of performance. While POMPDs have proven more useful than MDPs as representations of human cognition, they are notoriously challenging to solve in practical problem instances due to partial observability. The optimization of the POMDP policies almost never results in exact solutions; working with them requires a careful selection of a suitable approximation method. At the moment of writing, there is no one-stop solution available; rather, the best method depends on a number of factors and typically requires the attention of an ML engineer.

An exhaustive review of the methods is beyond the scope of this article. Nevertheless, we find the following attributes of POMDPs important to consider while choosing the policy optimization method (see also Kurniawati, 2022): (i) the size and structure of the state and action spaces, (ii) whether the state and action spaces are continuous or discrete, (iii) the nature of the components embedded within POMDPs (e.g., a neural network component), (iv) whether inference is needed online or offline, (v) the amount of computational resources available, and (vi) the size and structure of the observation space.

Available RL methods for policy optimization of POMDPs can be divided according to four dimensions relevant here: (i) model-free vs. model-based, (ii) on-policy vs. off-policy, (iii) deep vs. classical RL, and (iv) policy vs. value-based approaches. Model-free methods that include deep Q-Networks (DQN; Mnih et al., 2015), Proximal Policy Optimization (PPO; Schulman et al., 2017) and Q-learning (Watkins & Dayan, 1992) do not require an explicit model of the transition function, while model-based methods do. The benefit of a model-based approach such as Partially Observable Monte Carlo Planning (POMCP; Silver & Veness, 2010) is that planning (of actions) can be done directly using the model; however, learning the model for POMDPs remains an open problem, and these methods are slower to execute. On-policy methods such as actor-critic asynchronous advantage (A3C; Mnih et al., 2016) update policy during learning, while off-policy methods (e.g. PPO) collect rollouts offline and learn based on them. The best known classical method is Q-learning. This method is limited to small state-action spaces. Most work presently uses deep RL-based methods because of their superior generalizability and efficiency. Nevertheless, the use of deep-RL-based methods is a challenge due to their generally unstable behavior during training. Policy-based methods directly optimize the policy to maximize rewards and can handle continuous action spaces, while value-based methods which optimize the value functions tend to be preferred for discrete action spaces (Kurniawati, 2022).

### Reward Shaping

Reinforcement learning (RL) methods, both value-based and policy-based, aim to optimize an agent’s behavior to maximize the cumulative reward. However, in scenarios where the reward function is sparse and where few of the state transitions come with informative rewards, the process of identifying reward-maximizing behavior hinges on extensive stochastic exploration. Therefore, when the inherent reward structure for a task is sparse (e.g., primarily a terminal reward when the task is completed), it may be important to expedite learning for practical reasons. This may be achieved by introducing additional rewards to guide the RL process towards the optimal policy, by what is known as reward shaping. For instance, we might add a distance-based reward that focuses on exploration in parts of the policy space that are likely to offer good models of cognition.

Reward shaping techniques need to be implemented with careful consideration of how they impact agent behavior; small changes to reward functions can significantly change the optimal policy. One recommended mitigation mechanism is using a potential-based shaping term: suppose that we have a reward function $R(s,a,s')$ rewarding a transition from state *s* to $s'$ through action *a*. We could specify a shaping term $R_s(s,a) = \gamma \Phi (s') - \Phi (s)$ where $\gamma$ is the discount factor and $\Phi$ is an arbitrary function. It can be shown that the shaped reward $R(s,a,s') + R_s(s,a)$ has the same optimal policy as $R(s,a,s')$ (Ng et al., 1999). In the case of distance-based rewards discussed above, we could, for instance, define $\Phi (s)$ as the distance from the goal state, thereby rewarding the agent for getting closer to the goal and penalizing moving further away from it. The more common practice of ad hoc reward design is however typically “unsafe,” meaning that it leads to unintended or undesirable outcomes (Knox et al., 2023; Booth et al., 2023). A particular concern is reward shaping that is intended to increase efficiency but which changes the cognitive theory. Much like the practice of model building, designing more robust rewards can also benefit from an iterative workflow, and continuous refinement of the reward function, including with the help of methods that surfaces problematic edge cases where undesired behaviors are being incentivized by the shaped rewards (He & Dragan, 2021).

### Curriculum Learning

Just as humans do, RL algorithms sometimes struggle when faced with highly complex tasks. In many cases, it is possible to “kickstart” the learning by beginning with training from a simplified version of the task and gradually increasing the complexity of the task. This is called curriculum learning (Wang et al., 2021; Bengio et al., 2009). Curriculum design is essential for effective curriculum learning, yet there is little theory-based guidance in such a design at present. In general, it is best to adapt the curriculum to the speed at which the RL algorithm learns to perform these increasingly complex versions of the task, increasing the complexity only when it has learned to handle the current step in the curriculum.

### Hyperparameter Tuning

Hyperparameters—dropout rate, batch size, number of epochs, learning rate, etc.—are of practical importance because of their role in learning performance. To ensure that the model can adequately account for the data, we recommend careful hyperparameter tuning. The best option is to use an optimization method that, after enough trials, guarantees values approaching the optimum (e.g., Bayesian optimization). The next-best option is to rely on others’ values, from prior articles. Whatever the method of choice, open science practices require reporting of values that were used.

We trained the typing model’s policy with the goal of generalizability. The application goal was for it to cope with various keyboard designs and various individual-specific factors with links to cognitive capabilities (e.g., related to vision, finger agility, and working memory). To that end, the training process employed two loops: an outer loop randomly selects keyboard images and cognitive parameters, and an inner loop applies RL to learn the policy for optimizing the reward. For the algorithm, we chose Proximal Policy Optimization (Schulman et al., 2017), which offers a reasonable compromise in terms of practical implementation, sample complexity, and ready tuning.

To “boost” to the training, we employed reward shaping and curriculum learning. Because the traditional Boolean feedback for correct/incorrect typed text proved too sparse. The typing model uses the character-error rate for a distance-based reward, instead of a Boolean reward. This approach incentivizes progress towards typing the given text accurately. To facilitate initial learning, we could start with individual characters, then proceed to word level and ultimately advance to typing of phrases.

As for the tuning of hyperparameters, we began with settings informed by our baseline model (Jokinen et al., 2021a), for total timesteps, learning rate, and batch size. We then made greedy adjustments to these hyperparameters on the basis of the training-process convergence and average episode length.

## Parameter Fitting

The parameters of the internal environment are important in a computationally rational model because they can be adjusted to “fit” the model’s behavior to human data and generate predictions that approximate human behavior in a given situation, by the process known as parameter fitting or parameter inference. Parameter fitting is used to estimate the values of free parameters based on observed data and is not relevant for the parameters fixed to specific constant values during parameter specification. The fundamental assumption underlying a computational rationality model is that the policy controlling the internal environment is optimal. Accordingly, the policy (and its parameters) will be derived directly from the internal environment via an optimality condition. Adapting the model to reproduce human behavior is therefore done by manipulating the parameters defining the internal environment. This in turn results in a change in policy and consequently a change in predicted behavior.

Computationally rational models generally are not amenable to traditional parameter fitting techniques based on maximizing the likelihood of the observations (Myung, 2003). This is because (i) calculating the observations’ likelihood seldom falls to these models’ techniques and (ii) estimating it from model-output samples is generally infeasible due to the wide variety of behaviors possible. These conditions necessitate a different notion of model fit, often expressed in terms of deviation between what the model predicts and what was observed. To avoid calculating distances between high-dimensional data, it is a generally recommended practice to define this discrepancy in terms of the difference between statistics or other summaries calculated on both the predictions and the observations. This also lets us strip out irrelevant differences between the observations and predictions. Generally, any suitable distance or divergence function can serve to express the amount of deviation.

Various automated processes exist for fitting model parameters once the discrepancy between the model’s output and real-world observations has been captured. Manual fitting of the parameters in the traditional manner (trying out values until the model’s predictions “look like” the training data) might be tempting initially. However, this can turn out to be extremely labor intensive, especially in cases involving numerous parameters, and there is, in fact, no guarantee of finding the optimal values for them. Automated methods, on the other hand, do not suffer from these shortcomings.

Which automated methods are preferred? Given that mapping internal environment parameter values to the divergence evident in the model’s predictions involves optimization of the policy, calculating a gradient applicable to those parameters is impossible in most cases. Therefore, we confine our discussion here to gradient-free methods. The simplest potentially relevant automated technique is grid search: trying all possible combinations of parameter values and selecting the one that yields the smallest discrepancy. While this method does find an optimum, doing so is prohibitively expensive when the parameter space is large. Furthermore, parameters with a continuous domain require us to select an appropriate discretization, which is not necessarily straightforward. An alternative is to take an Approximate Bayesian Computation (ABC) approach. This refers to a large class of methods that can approximate a posterior over the model’s parameters with only sampling-level access to the model (Sunnåker et al., 2013; Aushev et al., 2023). Efficient implementations such as BOLFI (Gutmann & Corander, 2016) can quickly identify parameter values with a high posterior density. However, ABC is often too computationally intensive for real-time use. When we need rapid inference of the parameters, we can amortize the cost of these inferences. This is usually achieved via an ML model trained to predict the correct parameters for the model—i.e., the inference something like an ABC method would have drawn for the given set of observations (Moon et al., 2023).

The typing model implemented several internal parameters related to vision, finger action, and working memory. The vision module’s parameterization affects the speed of encoding, while finger-related values capture movement accuracy and the final set of parameters represents the uncertainty of the information held in working memory. To optimize the model for median human behavior, we fitted these parameters to the dataset. During the optimization process, we relied on the Jensen–Shannon divergence when designing the acquisition function. This measurement helps to accurately assess the distance at the distribution level between the simulation- and human-generated data. A shorter distance (less divergence) indicates greater similarity between the model and the human baseline.

## Model Checking

Model checking refers to qualitative and quantitative procedures that aid in verifying the validity of the models we build (Gelman & Shalizi, 2013; Mayo, 2018). It involves comparing model predictions with observed or expected data and examining the model in other respects. This is an important part of the iterative modeling workflow, and influences the decision to modify or accept models. Model checking is often carried out after fitting the model to empirical data, is fundamental for any process of iterative model development. Among the general classes of procedures available for model checking are (i) examining prediction accuracies and variances, (ii) graphically examining observed vs. predicted values, (iii) and turning to residuals, etc. for clues as to where the model is working well and where it is not.

### Prior and Posterior Predictive Checks

Model checking can begin as early as when we pick prior distributions on parameters or specify fixed parameter values. The prior predictive check involves simulating predictions based on these specifications (known as the prior predictive) and checking if they predict plausible data in a given experiment. If a prior predictive check fails, we return to the drawing board to re-specify the parameters or their priors so that they better align with our theoretical intuitions as well as prior beliefs about empirical data; we may even decide to change our the model architecture if we realize that our assumptions are not sound enough. Posterior predictive checks are similar, but involve simulations after observing the data (known as the posterior predictive), and carrying out parameter fitting or inference—checks here are about whether predictions based on the inferred parameters are able to predict the observed data at all (Baribault & Collins, 2023). If we are dealing with posterior distributions of parameters, predictions are generated by sampling from the posteriors and executing the model.

### Checks for Goodness of Fit

Model checking helps us assess goodness of fit, potentially identify model misspecification, find model refinement opportunities by detecting peculiarities in the empirical data, and compare models qualitatively. Several factors may lie behind invalidity or inaccuracy revealed by this step, such as (i) spurious or missing data, (ii) flaws in the model assumptions, (iii) inappropriate model structure, (iv) coding errors, (v) overfitting/underfitting for the data, and (vi) misapplication of theory. By iteratively refining and checking models, we converge to a plausible and well-scrutinized model that satisfactorily fits the data. This step can be carried out both qualitatively by visualizing model predictions against our data and examining the goodness of fit, and whether the data patterns of interest laid out earlier are captured by the model. Quantitative methods to assess goodness of fit often involve some measure of prediction error.

### Parameter Recovery Checks

Parameter recovery checks offer another powerful way to assess model behavior (Wilson & Collins, 2019; Heathcote et al., 2015). Having generated data from a known parameterization of the given model, one can evaluate whether the model fitting procedure chosen recovers the parameters originally used (or reproduces their influence). Researchers commonly generate visualizations that plot the parameter values estimated against a chosen range of “ground truth” parameter values. If these recovery plots indicate close correspondence between the two, the parameter recovery ability is deemed sufficient. Rigorous checking of parameter recovery dictates considering diverse sets of parameter values and examining any regions of the parameter space that seem particularly problematic. Among the tools available for this step are simulation-based calibration methods (Talts et al., 2018). By these and other means, parameter recovery checks support accounting appropriately for outliers and for issues such as any inherent temporal dependencies that the parameters should reflect.

### Ablation Studies

An ablation study tests the performance effects of removing one of the model’s theoretical constructs from the model (e.g., Kalman-filter-based belief updates where that is relevant). By conducting ablation studies, we can discern the significance and role of each component, thereby shedding light on their relative importance and potential interactions within the system. For example, we might want to ensure that the effects of working memory manipulation in experiments are linked back to the parameter that controls working memory size rather than an extraneous hyperparameter. Hence, ablation studies can help with model checking and further model improvement even when we are observing good model fits to produce models that are actually instantiating the theories we think they are instantiating.

### Outlier Analysis

Even if the model clearly passes the bar of the performance targets set, further tests are necessary, since no model is perfect. Human behavior almost always displays a long tail, as do models in some conditions. Accordingly, it is important to understand the causes of pattern of outliers. While it may be tempting to remove outliers, this is not good practice unless they can be definitively pinned to a specific confounding factor, such as participants misunderstanding the task.

We followed several model checking methods, for multifaceted evaluation of the touchscreen-typing model:

- We used existing empirical studies (Salvucci, 2001; Sarcar et al., 2016; Anderson et al., 1998) to establish a plausible range of parameter values for vision, finger, and working memory ($E_K \in [0, 0.05], F_K \in [0, 0.18], \lambda \in [0, 0.3]$). By assigning the range of values to these parameters and placing a uniform prior distribution over this range, we were able to capture a variety of plausible typing behavior that include both the fastest and slowest typing speeds for one-finger and two-thumb typing scenarios. The sensibility of the specified priors was verified using a prior predictive check using 100 independent typing episodes generated by random parameters from the prior. The average and median performance exhibited a right-skewed distribution, similar to what is expected in human data.

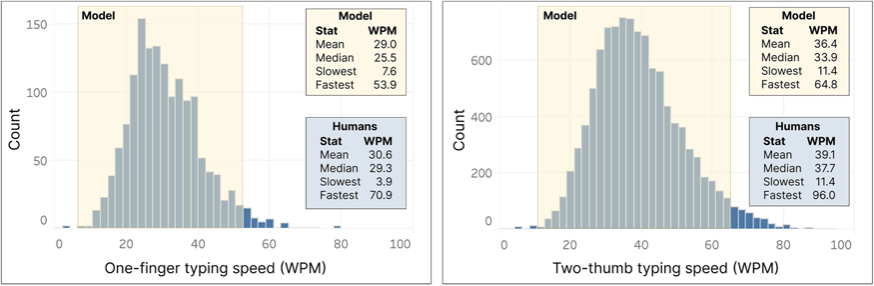

- After fitting parameters of our model to empirical data, we compared the model’s posterior predictions to data observed in our user study, i.e. a posterior predictive check, in each iterative step. To better judge its performance relative to human data, we employed words-per-minute values (shown in the prior predictive check figure above), inter-key interval, number of backspacing, error rate, number of gaze shifts, and gaze-on-keyboard time ratio as metrics (see Table 1). These simulated results can also be used to assess the model’s goodness of fit.
- Conducting an ablation study helped us evaluate the effectiveness of the working memory design. Our comparison entailed testing model designs that exclude a particular feature from working memory, to reveal the resulting impact on performance.
- Trajectory visualization gave us a better understanding of the model’s behavior by depicting both gaze and finger movements (as shown below, in panels a and b). The trajectories graphically illuminated patterns of gaze and finger movements during typing.
- We analyzed the model’s behavior under extreme parameter settings (for example, see panel c) to identify any anomalous behavior.
- In addition, we tested the model on unfamiliar keyboards (see panel d). The validation process assessed its ability to perform comparably on keyboards it had not seen before.

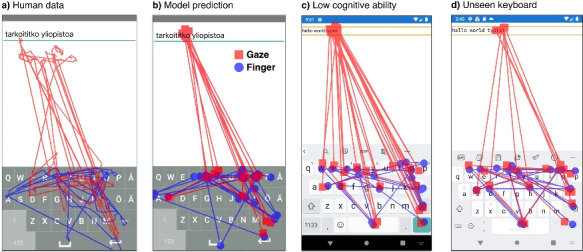

## Model Selection

Once model checking has filtered in at least one plausible candidate model, model selection becomes relevant. Model selection or model comparison refers to the process of comparing our candidate model(s) against each other and to other plausible models (e.g., the baseline models identified in earlier steps) to find the ones that best satisfy our modeling goals, given our data. It is a common practice to conclude that a model should be taken seriously because it performs well on model selection methods compared to competing models.

A common and important modeling goal is generalizability, so that any inference from modeling extends beyond the specific data samples and settings that it is fit to more generally applicable settings. Almost all quantitative model selection methods prioritize this goal and operationalize measures to assess generalizability. Cross-validation (Bates et al., 2023) is a method that assesses generalizability by leaving out data from the parameter fitting process and assessing the predictive performance of the fitted parameters on the held-out data. Given a set of models, the one that predicts held-out data the best is assessed as being the one that is most generalizable. Such assessment is useful, as it is otherwise easy to “overfit” models to random irrelevant fluctuations in the training data, consequently hindering the model’s ability to predict behavior accurately in new settings. Deep RL agents for instance are especially prone to overfitting (Zhang et al., 2018). One key aspect in implementing cross-validation is the choice of holdout sets when training and evaluating models of human behavior, especially the choice of study unit, i.e. are we holding out data at the level of participants, specific tasks, or individual trials? Ideally, we want models that generalize well at every level, the level of the participants and their individual tasks, the level of participants across tasks, the level of experimental groups, and the level of the population, but this may also depend on the inference goals. Variants of cross-validation also directly assess generalizability on a specific target domain instead of holding out data from the training distribution (Busemeyer & Wang, 2000).

Information criteria-based methods on the other hand prioritize generalizability without holding out data. They produce measures for preferring those models that best balance goodness of fit to observed data and simplicity (also known as Occam’s razor)—to avoid both overly simplistic “underfit” models and overly complex “overfit” models and find the model that has the best chance of generalizing to unobserved conditions. The information-criterion scores are a combination of a score that captures goodness of fit with another score that acts as a penalty for model complexity; these relative overall scores are often used to compare models, and the model with the lowest score is considered to be the “best” among the considered models. For example, the Bayesian information criterion is calculated as $BIC = -2 ln(\hat{L}) + k\times ln(n)$, and Akaike information criterion, $AIC = -2 ln(\hat{L}) + 2k$, where $\hat{L}$ is the likelihood estimate of the model, *k* is the number of parameters, and *n* is the sample size of the data; the first term is a measure of the goodness of fit, and the second is a penalty for model complexity measured as some function of the number of parameters. Being calculated differently, different model selection methods provide different results. There are several other information criteria-based methods, deviance information criterion (DIC), and Watanabe-Akaike information criterion (WAIC)—see Myung and Pitt (2018) each differing on how they calculate goodness of fit and penalize complexity.

Bayes factor (Kass & Raftery, 1995; Shiffrin et al., 2016; Schad et al., 2022) and minimum description length (Grünwald, 2007) are other methods that have been used in the literature. These scores can be more valid than information-criteria-based scores as they do not directly assume that the number of parameters in a model is reflective of complexity, but rather, also consider the structure of any prior distributions over parameters and the space of predictions the models make. However, these methods as well as cross-validation are computationally expensive, and information-criterion-based methods hence remain popular as a heuristic for estimating generalizability. As model selection methods use different heuristics to implement Occam’s razor, it is sometimes a practice to compare candidate models across several metrics and pick one that is favorable in most of the comparisons. This can be a good practice for transparency while selecting between models for standardized sets of tasks and contexts. However, we believe that ideally, the most appropriate statistical model selection method for the given setting should be emphasized after taking into consideration their advantages and shortcomings.

A context-dependent use of model selection methods is also needed because generalizability, while important is merely one potential goal of modeling. Other modeling goals can include aspects such as interpretability, causal consistency, estimation speed, and fairness which may or may not be related to generalizability (Bürkner et al., 2023; Dubova et al., 2024). It is also possible for these goals to trade off with each other. Thus, we believe that model selection is best treated as a multi-objective problem. Absent robust methods to evaluate models on a given set of goals with different utilities, a good practice would be to chart the relevant measurements yielded by models across the objectives, then analyze the tradeoffs that may exist, with our pre-defined goals for modeling in mind. A common adage in modeling is that “All models are wrong but some are useful” (Box, 1980); model selection in this perspective is about picking the model most useful for our given context.

**Table 1:** Comparison of model predictions against baseline predictions and observed data for one-finger typing

Metric	Human data	Baseline	CRTypist
Typing speed (WPM)	27.2 (3.6)	25.2	28.9 (4.4)
Interkey interval (ms)	381 (51)	399	366 (30)
Backspaces per sentence	2.6 (1.8)	1.5	2.4 (2.5)
Typing error rate (%)	0.6 (0.7)	0.5	0.1 (0.4)
Number of gaze shifts	3.9 (1.5)	4.2	5.5 (1.7)
Gaze on kbd (%)	70 (14)	87	71 (4)

**Table 2:** Assessing model performance on autocorrection and unseen keyboards

Novel keyboard settings	Metric	Human	Baseline	CRTypist
One-finger typing with auto-correct	Typing speed (WPM)	31.2	29	30.9
	Backspaces per sentence	2.46	0.1	3.2
One-finger typing on Gboard	Typing speed (WPM)	30.5	-	28.4
	Backspaces per sentence	2.0	-	3.6
One-finger typing on Swiftkey	Typing speed (WPM)	32.7	-	28.3
	Backspaces per sentence	2.1	-	3.7

The first step of model comparison in our case focused on contrasting the goodness of fit of the typing model against the baseline model (Jokinen et al., 2021a) for an empirical human-based typing dataset (Jiang et al., 2020). Our model’s estimates of typing speed (in words per minute, WPM), inter-key interval (IKI, in milliseconds), number of backspaces in a given sentence, typing error rate (percent of typed characters that are errors), number of gaze shifts to keyboard, and gaze-on-keyboard time ratio values all lay within one standard deviation of the humans’ data, and gaze shifts falls in two. Upon comparing our model’s performance to the baseline model’s, we concluded that ours did better on simple goodness of fit assessments on these measures. It outperformed the baseline model decisively by the last of these metrics, which the baseline model overestimated relative to the human data (see Table 1).

In addition to the goal of explaining the empirical data used for training well, we also cared about how well our model could adapt to unseen keyboard layouts. We trained the model on 28 keyboards and tested the model on 10 separate keyboards to evaluate its adaptability to them. When we used the model parameters optimized during training to predict performance on these keyboard layouts, we observed that the model performed well. It outperformed competing models on relevant metrics and showed comparable performance to the held-out data on the metrics (see Table 2 for an example of evaluations; the complete table for all goals and metrics can be found in Shi et al. (2024)).

## Discussion

The traditional POMDP framework provides an elegant way to describe interactive behavior. At its core are the decision-making entity (for example, the human participating in a behavior study), termed the agent, and the environment which constitutes everything external to the agent and which the agent interacts with (for example, the task in a behavioral study) (Sutton & Barto, 2018). While this distinction may seem straightforward and intuitive at first, often, the boundary between agent and environment is not identical to the physical separation between the two. The potentially tricky matter of appropriately delineating the agent relative to the environment in the context of the problem can be decisive for the agent’s eventual policy. Delineating between the notions of “internal” and “external” environments is similarly tricky. Different ways of distinguishing them can influence the model’s results considerably.

Consider the context of typing. Is the user’s finger a part of the external environment or, rather, the internal one? We could sensibly turn to perception for our boundary-setting conditions: the sensory experience of the user engaging with the screen via touch forms the limit of the internal environment, while the absolute location of the finger in space would be part of the external environment. Alternatively, we could situate the boundary in terms of the manner in which the agent receives rewards: is the reward signal directly determined by the external environment, constructed in the internal one, or conditioned on both? In our opinion, any strict rule for judging what falls within which environment is counterproductive. Instead, this design decision should consider the application context; domain expertise, attention to application scenarios, iterative development, solid testing, and the model selection procedures all play a role in helping determine the appropriate boundary. That said, many projects could benefit from the development of fine-grained principles that can inform the delineation between these environments in computational rationality settings but also specifying the agent–environment interface in RL operations.

### Human Adaptation, in Its Many Forms

After policy optimization, a computationally rational model predicts behavior that is optimally adapted to the fixed environment in which it was trained. Modeling should also consider that humans adapt continuously, however—when faced with an unfamiliar keyboard layout, people can slowly adapt their behavior to the new keyboard. Yet learning and adaptation have gone unexplored in computational rationality modeling, even though the theory of computational rationality implies that behavior should adjust in a computationally rational way. One reason is the raft of methodology-related challenges that accompany introducing a new level of complexity such that the model is optimally prepared to adapt its behavior to changes in the environment. Building and training of the model grow harder as the line blurs between adaptation occurring through policy optimization (primarily adjustment to fixed elements of the environment) and adaptation taking place after deployment (primarily adjustment to changing elements of the environments). One option, of course, is to make certain components of the model more elaborate, so that it captures the relevant human learning and habituation processes. A more machine-learning-oriented solution could use continual learning frameworks with neural networks and RL (Hadsell et al., 2020; Khetarpal et al., 2022). These have already begun to show encouraging results for handling distribution shifts that follow from changes in an agent’s environment. Nascent research that bridges computational rationality models with continual learning is making interesting inroads into exploring, alongside policy adaptation, changes in agents’ representation of the world as their experience grows in conditions of resource constraints (Arumugam et al., 2024). As for purely ML-oriented solutions, overparameterized neural networks are known to improve flexibility under covariate shift (Tripuraneni et al., 2021), and they may permit more adaptation to environmental changes when incorporated into neural-network-based components specified within the internal environment.

### Interactive Systems that Adapt to Human Users

The development of such applications as interactive AI systems faces a key challenge from the flipside of human adaptation: the system’s ability to adapt to the user. By approximating a person’s mental state and processes, computational rationality models can make a highly valuable contribution to adaptive interactive systems that strategically intervene to assist the user; for discussion, see the work of Mozannar et al. (2023). However, this is confounded by the recognized phenomenon of users following a mental model of the AI system. This model is geared for strategically steering the system towards user-desired behavior during interaction. While systems that comply with this steering may exhibit stronger interaction performance (Colella et al., 2020), a more advanced system could aim to identify and learn from users’ mental models of AI, their refinement over the course of the interaction, and the influence of mutable user goals on interaction behavior. Developing such mental models of AI systems is currently a research challenge, even more so in the context of learning these online during interaction (Howes et al., 2023; Steyvers & Kumar, 2022; Bansal et al., 2019). Co-operative multi-agent setups (Çelikok et al., 2019) with the user and the AI system as interacting agents are a promising approach to improve interactive behavior by better anticipating the user and their strategies—doing so with computationally rational user models would be an interesting avenue for further research (Howes et al., 2023); these are bound to be confronted by computational challenges in a real-time and interactive setting. Computational efficiency and approximation to computationally rational behavior can however be achieved by employing surrogate computational rationality models using methods such as amortized inference (Moon et al., 2023) or likelihood-free inference (Aushev et al., 2023; Palestro et al., 2018; Hartig et al., 2011).

### Balancing Model Complexity and Performance

Researchers need to balance the complexity of the internal environment with the accuracy of the simulation. Enhancing the model’s internal environment, when done right, can significantly improve the human-like behavior of the model, especially by simultaneously accounting for the many mental processes at play in learning, attention, memory, choice behavior, etc. However, the model then gets more complex, and building it correctly becomes more challenging. For example, in the case of the touchscreen-typing model, the current model has a simplified design that does not include reading behavior (Just & Carpenter, 1980) in its vision system. Neither does the module for working memory account for long-term memory (Norris, 2017), chunking (Yamaguchi & Logan, 2014), nor the impact of phrase sets. Incorporating these factors could afford valuable insight into intricate patterns of human behavior; however, tuning the hyperparameters of this type of model correspondingly can be immensely challenging. While one can use solid priors to simplify such procedures as setting parameters for the internal environment’s modules, building the model still may end up overly complicated. Hence, researchers may need to find the balance between model complexity and performance that are appropriate for their modeling goals and consequently often strive for the most realistic and satisfactory simulations without making the internal environment unduly complex.

### Optimization Approaches Other than RL

While we have concentrated here on RL-based mechanisms for generating boundedly optimal behavior, these are not the only conceivable means to that end. In fact, any approach that achieves optimal results is valid, including methods for black box combinatorial optimization (e.g., Sarcar et al., 2018). Naturally, the choice of methods affects which aspects of the modeling workflow matter most in the case at hand. For instance, the reward specification is crucial in RL but irrelevant in active inference. Our focus on the RL setting stemmed from a desire to contribute concretely to the state of the art: off-the-shelf solutions for implementing an optimal policy are widely available.

### The Relationship Between Data and Modeling

While the model development aspects of realistically predicting human behavior form the heart of our workflow, the aim, in the end, is to estimate observed data, and these are gathered in experiments. Any model, however sophisticated, is ultimately unproductive if the experiments and the measurements made are invalid, unreliable, or highly noisy. For successful inferences, the iterative modeling workflow must go hand in hand with a good workflow for experimentation. The two overlap somewhat in scope, as many experiments get informed by prior theory and model-based predictions. Therefore, they should together constitute a fundamentally iterative data-collection and model building system that enriches science.

## Data Availability

No datasets were generated or analyzed during the current study.

## Footnotes

1 Also known as resource rationality and algorithmic rationality.
